# Cottage Nurses and Their Training

**Published:** 1890-01-25

**Authors:** 


### Cottage Nurses and their Training.

### AN ABSTRACT FROM MISS BROADWOOD'S PAPER.

My sole right to deal with the subject consists in the fact that a system for benefiting the sick poor, which I was able, with the kind help of neighbours, to organise seven years ago around Ockley and Dorking, has proved so remarkably successful that it has been adopted around Reigate, Godalming, Haslemere, Lowestoft, Thetford, and many other places in England and Scotland. Persons of authority and knowledge have pronounced it the system best suited to cottagers, and the only one that consistently reaches them. That class of our poor when requiring a nurse are sometimes put to terrible shifts. I have known women tended in confinement by girls of only 13 or 14 years of age. Once it was "Caleb," a little lad of 12, whom I found the sole attendant of his mother and her new-born babe. She had had the foresight to instruct Caleb in gruel-making and other useful mysteries before she was laid up. Such cases, and still more another of a poor woman, with a child barely a week old, ruining her health over the family wash-tub, forced upon me strongly the truth of our doctor's repeated assertion that village nurses were needed.

Most country parishes being too poor or too thinly populated to employ any sort of nurse continuously, our plan has been to form an association of parishes limited to those within twelve miles of the secretary's home, and to maintain a staff of cottage nurses in the proportion of one for every 1,300 of the population. In each parish some lady is found to represent it on the committee of management; to her application is made by persons requiring a nurse; she collects and guarantees a sufficient annual contribution, and watches any case nursed within her parish. We are undenominational, and adopt the parish merely as a well-defined area in lieu of more convenient divisions. We have a sliding scale of subscriptions and fees. Thus, the labourers, subscribing 2s. annually, get a nurse for 2s. a week; artisans, who subscribe 3s., get a nurse for 3s. For small farmers and tradespeople the subscription and fees are 5s.; and for gentry 10s. To all non-subscribers the fees are doubled, as they are also for infectious cases; and, again, in a confinement, if no doctor is engaged, in order to discourage the employment of our nurses as midwives, they being only trained for *monthly* work. Board and lodging for the nurse must be provided in every case; but when a nurse is employed in especially poor cottages the association allows her 2s. 6d. a week towards extra board. The nurses lodge in different parts of the district, and each of them has a basket of appliances, and receives a dress and four aprons (not uniform), and a salary of from £20 to £28, but she must lodge herself and board herself when not out. We allow to each a clear month's holiday in the course of the year, and encourage saving by setting aside £1 for every year's services completed with the association, to which the nurses can, and mostly do, add by contributions not less than 10s. from their quarterly wages. We took for a basis of fair wages the fees charged by independent village nurses in our neighbourhood, which varied from 7s. to 10s. per week. Higher wages we cannot afford, with training to pay for, as each nurse brings us in only about £5 a year, because, being seldom able to attend more than one case at a time, she rarely gets through more than ten in her working year of eleven months. Cottage women of between twenty-one and forty are taken as probationers, and if they show care and interest in their work, and some competence in nursing easy cases of sickness, we give them training in the City of London or the British Lying-in Hospital. We have also found two months' work under the district nurses at Cambridge useful help to them, but one of these courses cost seven guineas and the other five guineas, and the expense of these greatly hampers our work. The three months' course given by Miss Lückes at the London Hospital for district nurses might benefit our probationers; but it costs £12, far more than our funds can afford. To St. Bartholomew's, owing to the kind instrumentality of Sir Dyce Duckworth, our nurses might have gone for a few months' instruction *gratis*; but the matron, Miss Stewart, after most kindly considering and discussing the matter, warned me that our nurses must mingle with the ladies who went for the short course, and, like them, assist at operations, which would probably awaken in them a taste for exciting surgical cases, such as they would not be called upon to tend in cottages. And I rest persuaded that, just as a course of cookery lessons with an elaborate *batterie de cuisine* would unfit the Indian cook for following his master into camp amongst hills and jungles, so a regular course of hospital training would spoil our homely cottage nurses for their vocation. It would be a wonderful help to us if each matron of a village hospital could be induced to fall in with some plan of training. Hitherto I have found them sadly disinclined to attempt it, and slightly jealous of "cottage nurses" lest they should keep the hospitals empty. The large London workhouse infirmaries might be even better schools for our probationers, because they possess maternity wards, and the training in the lying-in hospitals costs us seven guineas a nurse. But the trained lady nurses in charge of infirmaries to whom I made application, have hitherto refused help. Much they have learnt, but they are disinclined to impart a portion only of their knowledge. These lady matrons excused themselves to me, one and all saying, "a little knowledge was a dangerous thing," and that "no one who has not been through a three years' hospital course deserves the name of nurse!" Well, my idea is this. Ignorance is far worse than a little knowledge; and I may quote it as the opinion of Sir William Bowman, himself a trustee of the Nightingale Fund, that hospital-trained sick attendants have no right to arrogate to themselves the exclusive use of our good old English name of nurse, which was in use before any systematic organisation of hospitals or training was dreamt of. Let certificated nurses, with the aid of Greek and Latin lexicons, compound a distinctive appellation for the new species of womankind they represent, or, better still, let them generously recognise that they merely form the highest grades of the order to which all others belong who devote themselves to tending the sick and suffering. In every calling there are skilled and unskilled workers, and many amongst the unskilled who never expect to rise from their lower position. Now, when we find highly-skilled sick attendants endeavouring to establish trade rules by charter, and averse to instruct humbler women ready for rougher work than they themselves could stoop to, we are tempted to suspect a desire to shroud a very generally useful branch of knowledge in a magnificent but unnecessary cloud of mystery. It rests, gentlemen and ladies, with you and all concerned in the training of nurses in hospitals or infirmaries to induce these angels to descend from clouds that show signs of speedy dissolution, and to go to and fro on the face of the earth as ministers and dispensers of the art of ministering to the sick. The time seems approaching when the public who want sick nurses will clamour to have them classed and certificated according to the distinct fields of work for which they are suited, and demand that those of the higher grades should be competent and ready to instruct their humbler sisters of the craft. Some systematic classification and distinction is desirable, as the term "hospital-trained nurse" is altogether too vague to suit the distinctions which the public demand, and various sorts of nurses are required for various classes of patients and cases. But the sick cannot wait till nurses and their champions agree, and the cry from the friends of the cottagers increases. Not a week passes without applications reaching me asking if I can supply, or get training for, a cottage nurse; and I gladly take this opportunity of making more widely known my readiness and power to keep up a continuous supply of not less than two carefully-selected cottage pupil nurses to any workhouse infirmary or small hospital that will give a continual succession of three months' courses of the instruction we ask. Our probationers would, in return for their keep and teaching, undertake a daily portion of the rough housework of the institution, and I cannot but believe they might be found worth their salt. We will not stand aside, because we believe in our work; and we do not wish to stand still; but in moving forward we must be content to bring up the rear with our regiment of lower rank nurses—or call them what you will. They are an active part of the great army with which you and we and others are all fighting the miseries of birth, sickness, incurable disease, and death. We claim for this rearguard of the nursing army recognition, and some measure of training and encouragement; although its members may never get the opportunity of pushing to the front and gaining Victoria crosses.

---

The Chairman, Mr. Burdett, introduced the animated discussion which followed the reading of the paper by briefly stating the position occupied by the Hospitals Association in relation to nursing matters. This association appointed a committee to consider the question when, some time ago, an outcry was raised for the registration of nurses. This committee, having collected and considered the views of those engaged in the training of nurses throughout the country, considered it would be harmful and misleading to merely enter in a book the names of nurses who had had a certain amount of training, and issue a certificate, leading the public to suppose that anyone holding it was an efficient nurse, a suitable and eligible person for attending upon them in sickness. Though rules had been drawn up and a scheme prepared, the council decided to bow to the opinions of recognised authorities on this nursing question. There was still much said in favour of a scheme of registration which would entail on nurses—if he understood it—the payment of fees to a body outside the nurse's own school, and for the simple purpose that she might have one more piece of paper to commend herself to the public. If the public understood the nursing question they would know that every certificated nurse was a registered nurse, fully equipped for her duties, and selected after long trial. Taking, as he did, a deep interest in nurses and all that concerned them, he was opposed to the taking of a sum out of a nurse's small earnings for the purpose, as it was urged, of enabling her to earn a livelihood. If the nurse training schools, to whom they all owed so much for the position of skilled nursing at the present time, would issue an annual directory, with the name of every certificated nurse in this country, and the name of her school attached, this would be an act of justice to the nurses and a great benefit to the public at large. A person employing a nurse could consult this directory, a person having any complaint to make could send it to a particular training school, and there both the interests of the nurse and the interests of the public would be considered. It was, moreover, a hardship that nurses in many excellent small hospitals all over the country should not be certificated like their fellows in large hospitals. He would suggest that the authorities of the nurse training schools should institute examinations in certain country towns, admit to those examinations nurses who had been for three years working in small hospitals, and, if satisfied of their knowledge and capacity, grant them certificates. This would effect, he thought, all that was necessary in the interests of nurses and of the public.

Miss Wilson (secretary to the Workhouse Nursing Association) said that her ten years' experience went against the lines recommended by Miss Broadwood. Experience in a large number of cases was absolutely necessary for efficient training, and this could not be had in three months. In a small infirmary (fifty cases) not more than one case of typhoid or diphtheria, for instance, might be admitted in a whole year. How could such cases, when they occurred, be nursed by Miss Broadwood's cottage nurses? They had found if they lowered the training to less than a year the work suffered, and the doctors were not satisfied with the nurses. A nurse's first case, on going out after three months, might be entirely different from anything she had had to deal with in an infirmary during three months. Miss Broadwood would find it advantageous to spend more on efficient training, which would not in the least unfit her nurses for their particular work. Ladies did not suit for nurses in workhouse infirmaries, but suited a more superior class admirably. They got more authority over their patients, were less inclined to quackery, and had more chance of getting their orders carried out in a proper spirit.

Mr. Bonham-Carter (secretary of the Nightingale Fund) agreed with what had been said by Miss Wilson. A nurse ought to be trained for the most serious case, though no doubt good might be done even with very limited training. Still, it was really in a serious case that a nurse was required, and in such a case a person with only a few months' experience might make a grievous mistake. As to midwifery nursing, he did not think there had been any advance of late years in the skill which the ordinary country midwife showed; and he suggested that for the poor in country districts, and even in large towns, it might be better to start with making midwifery nursing the main object in view, and ordinary nursing a secondary object. The council of the Nightingale Fund, with the assistance of many medical men, had, fifteen years ago, made efforts for an improvement in this matter. The nurses were called, not midwives, but midwifery nurses, and six months' training in lying-in wards was fixed as a minimum, and there was a regulation that these nurses in country districts should work with the full support and consent of the medical men who were to superintend the work and be called in in case of danger. Unfortunately, the Nightingale Fund had not been able to take the matter up again, and establish training schools for nurses. Might it not be possible, with the assistance of an association, to make some provision to assist country districts in maintaining thoroughly-trained midwives? Miss Broadwood did not seem to refer to the practice of having nurses in the country to visit houses instead of residing there, and it was in this, he believed, lay the solution of one great difficulty.

Miss Glossop (a member of the Richmond Board of Guardians) thought the greatest difficulty must arise in a case contemplated in Miss Broadwood's paper, that of a nurse residing in a cottage where there were but two bedrooms and ten people. A nurse could not be sent to live there, but she might be of great assistance going in and out. She should think special cases which had been mentioned—fever, diphtheria, etc.—were better removed to hospitals, and in her own neighbourhood they encouraged the starting of infectious hospitals everywhere.

A Trained Nurse spoke of the Rural Nursing Association, which made midwifery nursing a chief object, supplemented by six months' training in hospital work. Might it not be better if Miss Broadwood's nurses were called cottage helps, for trained women knew that these were not trained nurses.

The Chairman, in closing the discussion, gave it as his opinion that three months in a hospital was practically not training at all. He agreed with Miss Paget's suggestion as to calling Miss Broadwood's nurses cottage helps. He was glad to announce a Christmas box or New Year's gift of an additional £10,000 from about 100 gentlemen, collected without one penny of expense, to the Pension Fund, to augment the donation bonus fund, already amounting to £26,000.

Dr. Steele (superintendent of Guy's Hospital) whilst admitting that anything less than a year's training was not sufficient, contended that Miss Broadwood's nurses supplied a real want amongst the poor in country districts.

Votes of thanks were passed to the authorities of Westminster Hospital for the use of their board-room, and to the chairman for presiding, Mr. P. Michelli, Sir Wm. Moore, and Mr. Bonham-Carter speaking to the resolutions.

